# The History and Capabilities of Herbal Simples

**Published:** 1891-05-23

**Authors:** W. T. Fernie


### THE HISTORY AND CAPABILITIES OF HERBAL SIMPLES.

By W. T. FERNIE, M.D.

### XXXI.—THE ELDER.

THE common elder, says Gerarde, groweth everywhere; it is planted about Cony boroughs, for the shadow of the conies. Formerly it was much cultivated near our English country cottages, because supposed to afford protection against witches. Hence it is that elder trees may be so often seen in the immediate vicinity of old village houses. It acquired its common name from the primitive word "eller" or "kindler," because its hollow branches were used to blow a fire into brightness. Sometimes the elder is called bore tree, or bour tree, from the central pith in the younger branches, which children bore out to make pop-guns. The botanical name—Sambucus nigra—has derived its substantive appellation from *sambukee*—a sackbut; in making the pipes of which, and of other musical instruments, the young branches of the elder, with their pith removed, were much employed. This tree was the "*aktee*" of the Greeks, commended by Dioscorides and Galen. Also Hippocrates gave its bark as a purgative; and from his time the whole tree has possessed a medicinal celebrity. German writers have declared it contains within itself a magazine of physic, and a complete chest of medicaments. Its adjective "nigra"—black—refers to the colour of the berries. On the same principle I once heard the worshipful Mayor of Plymouth, in a public speech, call the Niger Expedition the "nigger" expedition. Belonging to the order of Caprifoliaceous (with leaves eaten by goats) plants the elder bush grows to the size of a small tree, having many white flowers in large flat terminal umbels. It exhales an unpleasant soporific smell, which is said to prove harmful to those who sleep under its shade Our summer is never fully here until the elder is in flower; and it ends when the berries are ripe. In floral language the plant symbolises "zealousness." The leaves when bruised and worn in the hat, or rubbed on the face, will prevent flies from settling on the person. But few insects will remain in any spot over which an infusion of the leaves has been sprinkled. If turnips, cabbages, fruit trees, or corn (which are subject to blight from a variety of these assailants) are whipped with the branches and green leaves of elder, they will gain an immunity from all such depredations. Sheep which have the rot, if able to get at the bark, and the young shoots, will thus cure themselves of this affection. Mice will forsake granaries where the green plant has been introduced. "The leaves of elder boiled soft, and with a little linseed oil added thereto, if then laid upon a red cloth, or piece of scarlet, and applied to piles as hot as this can be suffered, being removed when cold, and replaced by one such cloth after another upon the diseased part, by the space of an hour, and in the end some bound to the place, and the patient put warm to bed—this hath not as yet failed at the first dressing to cure the disease; but if the patient be dressed twice, it must needs cure them if the first fail." Chemically, the flowers contain a yellow odorous buttery oil, with tannin, and malates of potash and lime. The berries furnish viburnic acid. On expression they yield a fine purple juice, which proves a useful laxative and a resolvent in recent colds, as well as in sundry chronical disorders. A medicinal tincture is made from the fresh inner bark of the young branches. This, when given to excess, will induce profuse sweating, and will cause asthmatic phenomena. When used more skilfully, it is highly beneficial for the same conditions if occurring as disease—particularly for the spurious croup of children, which wakes them at night with a suffocative cough and wheezing. A dose of two or three drops if given then, and perhaps repeated in fifteen minutes, will quickly prove of singular service. The same medicine will relieve neuralgic pains in the forearms. Sir Thomas Browne said that in his day the Elder had become a famous medicine for quinsies, sore throats, and strangulations. The inspissated juice, or "rob," extracted from the crushed berries, and simmered with white sugar, is cordial, aperient, and diuretic. This was a popular early English remedy taken hot at bedtime when a cold had been caught. One or two tablespoonsfuls should be had for a dose with a tumblerful of very hot water. It promotes perspiration, and is demulcent to the chest. Five pounds of the berries are to be used with one pound of loaf sugar, and the juice should be evaporated to the thickness of honey. Also a capital wine is commonly made in this country from the berries, with raisins and sugar and spices. This is a famous domestic cordial, and may pass well for Frontignac. When well brewed, and three years old, it constitutes English port. A cup of mulled elderwine, served with sippets of toast and grated nutmeg, just before going to bed on a winter night, is a thing—as Cobbett would say—the be "run for." Its "cinnament, ginger, nutmeg, and cloves" may have had much to do with the "jolly red nose" of Simon the Cellarer, in the good old song commemorative of that worthy. The Romans made use of the black elder juice as a dye for the hair. From the flowers a fragrant water is distilled as a perfume; and a gently stimulating ointment is prepared, with lard for treating burns and scalds. Another ointment made from the green berries with camphor and lard is ordered by the London College, and is curative of piles. The juice of elder root, when taken in a dose of one or two tablespoonsful while fasting, acts as a strong aperient, being the most excellent purger of watery humors in the world, and very singular against the dropsy if taken once a week. Gerarde tells us, "The gelly of the Elder, otherwise called 'Jew's ear,' taketh away inflammations of the mouth and throat if they be washed therewith, and doth in like manner help the uvula." He probably refers here to a fungus bearing the same name, and resembling in shape the human ear, which grows often from the trunk of the elder. This is the tree upon which the legend represents Judas as having hanged himself, or of which the cross was made at the crucifixion. Alluding to this fungus, or moss, and to the supposed fact that the berries of the elder are poisonous to peacocks, a quaint old rhyme runs thus—

"For the coughe take Judas' Eare,
With the paring of a Peare;
And drynke them without feare,
If you will have remedy.

"Three syppës for the hycocke,

And six more for the chycocke;

Thus will my pretty pycocke

Recover by-and-by."

(*To be continued.*)

